# Comparison of clinical outcomes between culture-positive and culture-negative sepsis or septic shock pediatrics patients: A systematic review and meta-analysis

**DOI:** 10.5339/qmj.2024.32

**Published:** 2024-07-29

**Authors:** Rahil Khowaja, Fazila Karimi

**Affiliations:** 1 School of Medicine, Swansea University, Swansea, United Kingdom *Email: Khowajarahil16@gmail.com; 2 School of Public Health, SZABIST University, Karachi, Pakistan

**Keywords:** Pediatric, culture-negative, culture-positive, sepsis or septic shock, meta-analysis

## Abstract

**Introduction:**

Comparatively, culture-negative septic shock or septic shock (CNSS) is frequently observed among pediatric patients, contrasting with the more distinct clinical profile and prognosis of post-surgical septic shock (CPSS). However, limited data are available on the outcomes of CNSS in comparison to CPSS in pediatric patients. This study seeks to conduct a systematic review and meta-analysis of existing literature to comprehensively compare outcomes between CNSS and CPSS in pediatric patients.

**Methods:**

Electronic databases, such as PubMed, CINAHIL, and EMBASE, were systematically searched up to January 15, 2024, using predefined terms. We included all studies that compared outcomes between CPSS and CNSS in pediatric patients. The primary outcome evaluated in this study was all-cause mortality. Secondary outcomes included length of hospitalization, length of intensive care unit (ICU) stay, and duration of mechanical ventilation (all measured in days).

**Results:**

Among the initially identified 1328 articles, six studies involving 2511 pediatric patients met the inclusion criteria and were part of this meta-analysis study. The pooled analysis revealed no significant differences in all-cause mortality (odds ratio: 1.26, 95% confidence interval (CI): 0.93 to 1.70, *p* = 0.14), length of ICU stay (mean difference (MD): 0.18, 95% CI: -0.33 to 0.68, *p* = 0.50), and duration of mechanical ventilation (MD: -0.74, 95% CI: -2.46 to 0.98, *p*-value = 0.40) between CPSS and CNSS. However, the length of hospital stay was longer in CPSS compared to CNSS (MD: 7.38, 95% CI: 5.50 to 9.27, *p* < 0.0001).

**Conclusion:**

Approximately 26.56% of pediatric septic cases were culture-positive. There were no statistically significant differences in mortality, ICU stay, and duration of mechanical ventilation between CPSS and CNSS. However, hospital stay was prolonged by more than 7 days in culture-positive cases. Further multicenter studies are warranted to validate these findings and explore additional presentation characteristics.

## Introduction

Sepsis stands as a prominent cause of death, illness, and increased utilization of healthcare among children globally.^1^ The mortality rate for children affected by sepsis varies widely, ranging from 4% to 50%.^2^ This variation depends on factors like geographic location, risk factors, disease severity, and available resources.^3^ In the management of septic children experiencing multiple organ dysfunction syndromes and/or resistant shock, treatment typically involves organ function replacement therapy, including renal replacement therapy and/or mechanical ventilation.^4^

Previous studies have underscored the importance of identifying pathogens, providing crucial information for selecting appropriate antibiotics, and resulting in improved prognoses.^5,6^ In the subset of patients encountering septic shock, there exists a subgroup characterized by an unidentified causative pathogen in culture, known as culture-negative septic shock (CNSS). Despite estimates suggesting CNSS prevalence among all septic shock cases to range between 30% and 50%, the complete understanding of its associated clinical features remains elusive.^7^ Differences between CNSS and culture-positive septic shock (CPSS) in terms of epidemiology, pathogenesis, and effectiveness of treatment plans may potentially exist.^8^

Although a meta-analysis involving 10 studies among adult patients showed no statistically significant variances between CPSS and CNSS cases regarding outcomes such as all-cause death, mechanical ventilation requirements, requirements for renal replacement therapy, and intensive care unit (ICU) stay duration, a noteworthy divergence was observed in the average duration of hospitalization. Culture-positive cases notably exhibited a statistically significant prolonged hospital stay compared to culture-negative cases (mean difference [MD]: 3.04, 95% confidence interval [CI]: 2.25–3.82, *p*-value < 0.001).^9^

While numerous studies have explored the clinical outcomes and characteristics of CNSS and CPSS in adult patients,^9^ data for pediatric patients remain limited. Therefore, this meta-analysis aims to compare the characteristics and outcomes of pediatric patients with CPSS and CNSS. Additionally, the comparison of CPSS and CNSS in pediatric patients has been inadequately addressed in the existing literature, with only a few studies hindered by small sample sizes. Hence, this meta-analysis was performed to compare clinical outcomes between CPSS and CNSS pediatric patients.

## Materials and Methods

This study followed the “Preferred Reporting Items for Systematic Reviews and Meta-Analyses (PRISMA)” guidelines. The protocol of this meta-analysis was registered with PROSPERO (CRD42024505689).

### Search strategy

Two authors performed a comprehensive searching in online databases like PubMed, CINAHL, and EMBASE, spanning from the inception of databases to January 15, 2024. Additionally, we also searched Google Scholar to find additional studies. The search terms included “culture-positive,” “culture-negative,” “sepsis,” “severe sepsis,” and “septic shock.” Medical Subject Headings (MeSH) terms were employed in conjunction with synonyms. Adjustments were made to the search terms for each database (Supplementary Table 1). Additionally, we examined the bibliographies of the included studies to find any study relevant to the study topic. Any disagreement between the two authors was resolved through discussion.

### Study selection

We included studies in this meta-analysis, if the following inclusion criteria were met: (1) including pediatric patients (under 18 years old) with sepsis or septic shock. We excluded studies with unquantifiable data and those lacking clear outcome comparisons. Additionally, letters and reviews were excluded from the analysis. Studies involving adult patients (age >18 years) were also not considered. We also excluded studies that lacked a comparison group. We imported all studies into the EndNote X9 version. Following the removal of duplicates, the first-level screening of included studies was conducted based on abstracts and titles. Subsequently, the complete texts of eligible records were acquired, and a thorough assessment was performed using pre-established eligibility criteria. Any discrepancies in the process of study selection were resolved via discussion.

### Data extraction and outcome measures

We collected data from the studies included by utilizing a data extraction sheet created with Microsoft Excel. The extracted data encompassed various parameters, including the author’s name, publication year, region where the study was conducted, sample size, and outcome metrics. The primary focus was on all-cause mortality, which included mortality rates within hospital settings as well as those at 28 and 90 days post-intervention. Secondary outcomes comprised the ICU stay duration, hospital stay duration, and the duration of mechanical ventilation (all measured in days). In case of missing data about the outcome, an email was sent to the corresponding author of the included studies. Two authors extracted data independently, and any disagreement was resolved through discussion.

### Quality assessment

Two investigators independently performed a quality assessment. The Newcastle-Ottawa Scale was used for this purpose. This scale, with a maximum score of nine points, assesses the selection, comparability of groups, and outcomes within cohort study populations. Based on this assessment, the studies were classified as poor (0 to 3 points), fair (4 to 6 points), or good (7 to 9 points) in terms of their quality.

### Statistical analysis

We used Review Manager Version 5.4.1 for statistical analysis. For dichotomous outcomes, we reported the odds ratio (OR) with a 95% CI, and for continuous outcomes, we presented the MD with a 95% CI. We used *I*-square and Cochran *Q*-statistics to compute heterogeneity among the study results. A *p*-value <0.1 or *I*^2^ ≥50% showed significant heterogeneity. We used a random effects model to deal with heterogeneity among the study results. Some continuous variables, initially reported as median with interquartile range, were transformed to mean as well as standard deviation for meta-analysis.^10^ A significance threshold of *p* < 0.05 was applied. As the total studies were less than ten, an evaluation for publication bias was not feasible.

## Results

**Figure 1.**
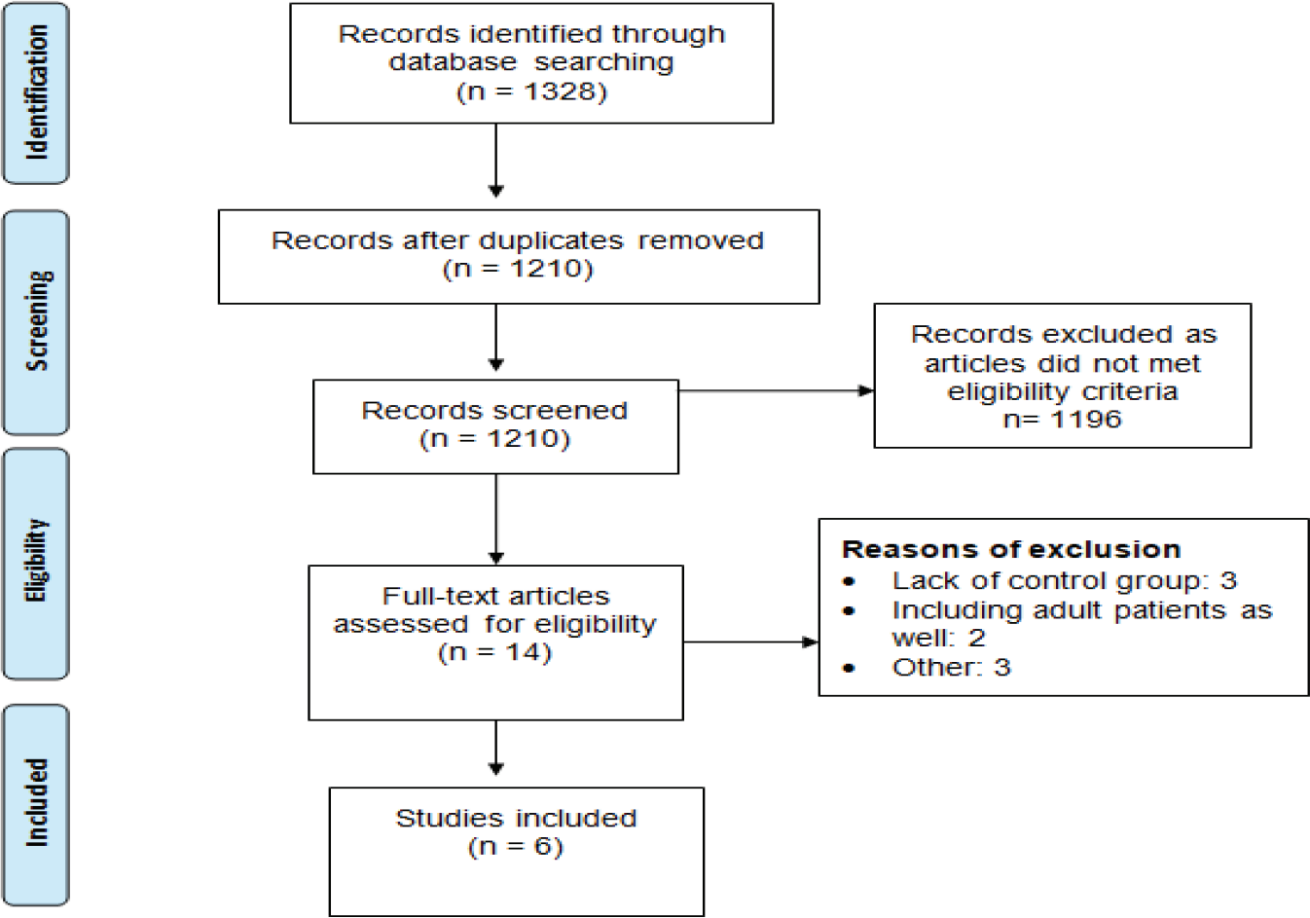
Flowchart depicting the selection process of studies following the PRISMA guidelines.

Figure 1 depicts the study selection process. The initial database searches identified 1328 articles. Following duplicate removal, an initial screening of 1210 articles was carried out based on abstracts and titles, adhering to predetermined inclusion criteria. Full texts of all eligible articles (*n* = 14) were obtained, ultimately resulting in the inclusion of six studies in the present meta-analysis.

### Study characteristics

Table 1 presents the characteristics of the studies included in this meta-analysis. The included studies were published between 2020 and 2023. Among these, three were carried out in the United States, with one each in Korea, China, and Saudi Arabia. The proportion of pediatric patients diagnosed with CPSS was 26.56% (667 out of 2511 patients). However, there was notable variation across the included studies, ranging from 8.95% to 62.92%. The assessment of the risk of bias is provided in Supplementary Table 2, indicating that all studies were deemed to be of high quality.

### Primary outcome (all-cause mortality)

**Figure 2.**
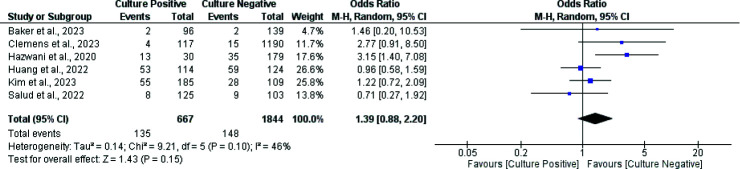
Forest plot comparing all-cause mortality between CPSS and CNSS pediatric patients.

Six studies, involving 2511 patients, assessed the risk of all-cause death. The overall rate of all-cause death was approximately 11.27% (*n* = 283). Among these, the CPSS had a mortality rate of 20.23% (*n* = 135), while the CNSS had a rate of 8.03% (*n* = 148). We did not observe any significant difference between the two groups in all-cause mortality between the culture-positive and culture-negative groups (OR: 1.26, 95% CI: 0.93 to 1.70, *p*-value: 0.14, *I*-square: 46%) (see Figure 2).

### Secondary outcomes

#### ICU length of stay (days)

**Figure 3.**
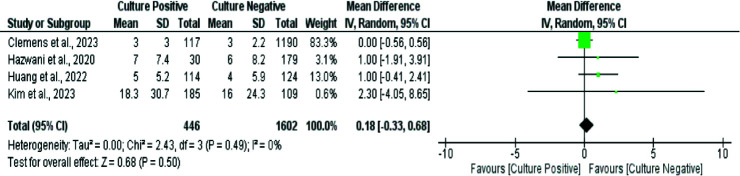
Forest plot comparing the mean length of ICU stay between CPSS and CNSS pediatric patients.

Four studies evaluated the ICU stay duration between CPSS and CNSS patients. No statistically significant difference was observed in the ICU length of stay between the two groups (MD: 0.18 days, 95% CI: -0.33 to 0.68, *p*-value: 0.50, *I*-square: 0%) (see Figure 3 for details).

### Hospital length of stay (days)

**Figure 4.**
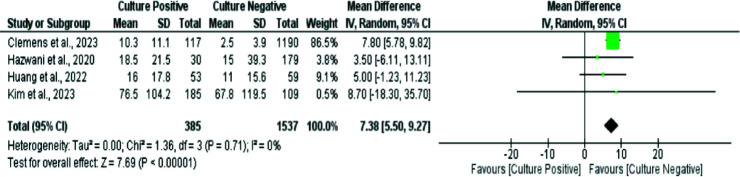
Forest plot showing comparing hospital stay duration between CPSS and CNSS pediatric patients.

Four studies also compared the duration of hospital stays between the CPSS and CNSS groups. Patients with culture-positive had a significantly longer hospital stay compared to those in the culture-negative group (MD: 7.38 days, 95% CI: 5.50 to 9.27, *p*-value < 0.0001, *I*-square: 0%) (Figure 4).

### Duration of mechanical ventilation (MV) (days)

**Figure 5.**
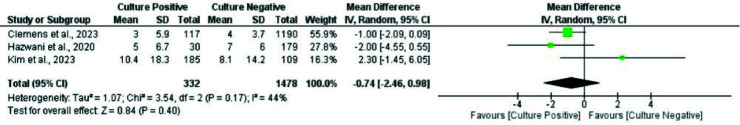
Forest plot comparing mechanical ventilation duration between CPSS and CNSS pediatric patients.

Three studies were incorporated in analyzing the duration of MV between CPSS and CNSS. The duration of MV was higher in patients with CNSS compared to CPSS. However, the difference was statistically insignificant (MD: -0.74 days, 95% CI: -2.46 to 0.98, *p*-value: 0.400.06, *I*-square: 44%) (Figure 5).

## Discussion

The current meta-analysis, comprising six studies and involving 2511 pediatric patients, examined the comparison between clinically positive and clinically negative cases of sepsis or septic shock. The combined analysis revealed that only approximately 26.56% of patients exhibited a culture-positive infection. Furthermore, the pooled analysis indicated no significant difference between patients with culture-positive and culture-negative sepsis or septic shock in terms of all-cause mortality.

Several factors contribute to patients presenting with culture-negative sepsis or septic shock. For instance, some patients may have already received antibiotics at local healthcare facilities prior to blood culture collection. Additionally, hospitalized patients may have been administered antibiotics before the onset of sepsis.^16^ Individuals with weakened immune systems, such as those with viral or fungal infections, may exhibit symptoms resembling sepsis.^17^ Additionally, some patients may show septic shock symptoms due to non-infectious conditions like inflammatory diseases and hematological malignancies.^18^

Apart from all-cause mortality, there were no significant disparities observed between the two groups concerning ICU length of stay and duration of mechanical ventilation. Nonetheless, patients with culture-positive sepsis exhibited a prolonged overall hospital stay compared to their counterparts. These distinctions may be attributed to variations in patient demographics, bacterial resistance to antibiotics, and the distribution of infection sites. A meta-analysis conducted by Li et al.^19^ yielded analogous results, indicating no notable distinction in the need for renal replacement therapy between culture-positive and culture-negative groups. However, due to insufficient data in the studies analyzed in this meta-analysis, we were unable to evaluate this outcome.

One potential reason for the extended hospital stay observed in the culture-positive group could be the necessity for a prolonged course of antibiotics, especially in scenarios involving abscesses, bacteremia, and osteomyelitis. Two studies^4,14^ within the analysis provided data on the duration of renal replacement therapy, revealing no notable distinctions between the two groups. Furthermore, the pooled analysis of three studies demonstrated no significant variance in the duration of mechanical ventilation between the culture-positive and culture-negative groups, suggesting similar requirements for organ functional support in both cohorts.

Past studies that included adult patients and compared the clinical characteristics between CNSS and CPSS have shown contradictory results. Effective utilization of antibiotics, as per the identified pathogen, can have a positive impact on clinical progress.^20,21^ For instance, a study conducted by Gupta et al.^22^ reported increased mortality in CNSS patients, attributing this finding to the precise information about the cause of their condition, allowing them to receive appropriate antibiotics. Conversely, a study by Yang et al. found poor outcomes in patients with CPSS, attributing the high mortality rate to distinctions in characteristics of patients, distribution of infection sites, delayed initiation of antibiotic treatment, and sociodemographic factors.^23^

The findings of this meta-analysis, alongside previous studies, highlight the significance of CNSS among pediatric patients. The studies included in this review underscore the challenge of distinguishing patients with negative and positive cultures during their initial presentation.^4,11,14^ Consequently, the International Pediatric Sepsis Consensus Conference emphasizes the early administration of antibiotics and fluid boluses to all patients with septic shock.^24^ A blood culture remains a crucial component of the diagnostic assessment of sepsis and septic shock, offering valuable guidance for targeted antibiotic therapy when positive. However, there is limited data available assessing the distinctions in the presentation of CPSS and CNSS, such as laboratory measures. Among the six included studies, only two compared electrolytes like procalcitonin, sodium, potassium, and so on^4,12^ between the two groups. Both studies found significantly higher levels of procalcitonin in patients with a positive culture compared to their counterparts, suggesting the potential utility of laboratory tests in developing predictive tools for the identification of children at greater hazard of systemic infection. Additionally, platelet levels were lower in the culture-positive group. However, certain parameters, including blood tests and the qSOFA score at baseline, were not consistently compared between CPSS and CNSS in the majority of included studies. Future studies comparing these laboratory values between the two groups at the time of presentation are needed to validate the usefulness of these measures in identifying children at increased risk of septicemia.

The current meta-analysis has certain limitations. Firstly, the number of studies included in the pooled analysis is small. Therefore, we need future studies with a large sample size to confirm these findings. Despite the inclusion of diverse cohort studies, significant heterogeneity persisted among them. Both retrospective and prospective cohort studies encompassed highly heterogeneous populations. Another consideration is that the suspected source of infection could serve as a key effect modifier, as certain infection sources exhibit increased rates of culture positivity and low death rates (particularly urinary tract infections), while others show low culture positivity rates and high death rates (especially pulmonary infections). It is worth noting, though, that not all the studies specified the suspected infection source. Lastly, characteristics at presentation were not comprehensively assessed by most of the studies. It is important to understand how the initial presentation varies between the two groups to take the necessary measures until the blood culture report has arrived. We need future studies to understand how outcomes like the requirement of vasopressors, renal replacement therapy, and organ dysfunction vary between the CPSS and CNSS groups.

## Conclusion

A culture-positive infection was present in 26.56% of children with sepsis or septic shock, according to the meta-analysis. Regarding the length of mechanical ventilation, length of ICU stays, and all-cause mortality, no statistically significant differences were found between the two groups. However, patients with positive cultures had a significantly longer hospital stay compared to those with negative cultures. It is necessary to do more multicentric research to confirm these results and investigate how the presentation traits of the culture-positive and culture-negative groups differ.

## Authors’ Contributions

RK: study design, study searching, study selection, quality, assessment, data analysis, and manuscript drafting; FK: study design, study searching, study selection, and manuscript drafting. The final version of the meta-analysis was reviewed by both authors.

## Data Availability Statement

All data generated or analyzed during this study have been included in the published article.

## Conflict of Interest Statement

The authors affirm that they do not have any competing interests.

## Figures and Tables

**Table 1.** Characteristics of studies comparing CPSS and CNSS pediatric patients.

			**Culture**	
**Study ID**	**Year**	**Region**	**Positive**	**Negative**
Baker et al.^11^	2023	United States	96	139
Clemens et al.^12^	2024	United States	117	1190
Hazwani et al.^13^	2020	Saudi Arabia	30	179
Huang et al.^4^	2022	China	114	124
Kim et al.^14^	2023	Korea	185	109
Salud et al.^15^	2022	United States	125	103

**Supplementary Table 1.** Search strategy for databases.

**Database**	**Search strategy**
PubMed	((culture negative sepsis OR culture negative septic shock AND (english[Filter])) AND (culture positive sepsis OR culture positive septic shock AND (english[Filter]))) AND (pediatrics OR children AND (english[Filter]))
EMBASE	(((“culture negative sepsis” OR “culture negative septic shock”) AND (english[Filter])) OR ((“culture positive sepsis” OR “culture positive septic shock”) AND (english[Filter]))) AND ((pediatrics/exp OR (child OR children OR adolescent OR neonate))/exp OR ‘infant’/exp) AND (english[Language])
CINAHIL	((TI OR AB OR KW: “culture negative sepsis”) OR (TI OR AB OR KW: “culture negative septic shock”)) AND (TI OR AB OR KW: “culture positive sepsis”) OR (TI OR AB OR KW: “culture positive septic shock”)) AND (TI OR AB OR KW: “pediatrics”) OR (TI OR AB OR KW: “children”) AND (english[Language])

**Supplementary Table 2.** Risk of bias assessment of included studies.

**Study ID**	**Selection**	**Comparison**	**Exposure or outcome assessment**	**Overall**
Baker et al.^11^	4	2	3	Good
Clemens et al.^12^	4	2	2	Good
Hazwani et al.^13^	3	1	2	Good
Huang et al.^4^	4	1	3	Good
Kim et al.^14^	4	2	3	Good
Salud et al.^15^	3	2	2	Good

## References

[1] Ranjit S, Kissoon N (2021;). Challenges and Solutions in translating sepsis guidelines into practice in resource-limited settings. Transl Pediatrics.

[2] Weiss SL, Peters MJ, Alhazzani W, Agus MS, Flori HR, Inwald DP (2020;). Surviving sepsis campaign international guidelines for the management of septic shock and sepsis-associated organ dysfunction in children. Intensive Care Med.

[3] Fernández-Sarmiento J, De Souza DC, Martinez A, Nieto V, López-Herce J, Soares Lanziotti V (2022;). Latin American consensus on the management of sepsis in children: Sociedad latinoamericana de cuidados intensivos pediátricos [Latin American Pediatric Intensive Care Society] (SLACIP) Task Force: Executive summary. J Intensive Care Med.

[4] Huang H, Chen J, Dang H, Liu C, Fu YQ (2022;). Comparing outcomes between culture-positive and culture-negative septic shock in a PICU: A retrospective cohort study. Front Pediatrics.

[5] Scheer CS, Fuchs C, Gründling M, Vollmer M, Bast J, Bohnert JA (2019;). Impact of antibiotic administration on blood culture positivity at the beginning of sepsis: A prospective clinical cohort study. Clin Microbiol Infect.

[6] Garnacho-Montero J, Garcia-Garmendia JL, Barrero-Almodovar A, Jimenez-Jimenez FJ, Perez-Paredes C, Ortiz-Leyba C (2003;). Impact of adequate empirical antibiotic therapy on the outcome of patients admitted to the intensive care unit with sepsis. Crit Care Med.

[7] Phua J, Ngerng WJ, See KC, Tay CK, Kiong T, Lim HF (2013;). Characteristics and outcomes of culture-negative versus culture-positive severe sepsis. Crit Care.

[8] Rangel-Frausto MS, Pittet D, Costigan M, Hwang T, Davis CS, Wenzel RP (1995;). The natural history of the systemic inflammatory response syndrome (SIRS): A prospective study. JAMA.

[9] Afzal MS, Chennuri RN, Naveed H, Raveena Bai B, Hanif R, Shahzad Z (2023;). Comparison of clinical outcomes between culture-positive and culture-negative sepsis and septic shock patients: A meta-analysis. Cureus.

[10] Wan X, Wang W, Liu J, Tong T (2014;). Estimating the sample mean and standard deviation from the sample size, median, range and/or interquartile range. BMC Med Res Methodol.

[11] Baker AH, Leland SB, Freiman E, Herigon JC, Eisenberg MA (2023;). Characteristics and outcomes of culture-positive and culture-negative pediatric sepsis. J Pediatrics.

[12] Clemens N, Wilson PM, Lipshaw MJ, Depinet H, Zhang Y, Eckerle M (2024;). Association between positive blood culture and clinical outcomes among children treated for sepsis in the emergency department. Am J Emerg Med.

[13] Hazwani TR, Kazzaz YM, Alsugheir S, Aldelaijan S, Alsugheir F, Alali H (2020;). Association between culture-negative versus culture-positive sepsis and outcomes of patients admitted to the pediatric intensive care unit. Cureus.

[14] Kim DH, Park SJ, Jhang WK (2023;). Comparison of the clinical characteristics and clinical outcomes of culture-positive septic shock and culture-negative septic shock among pediatric patients. PLoS One.

[15] Salud D, Reeder RW, Banks RK, Meert KL, Berg RA, Zuppa A (2022;). Association of pathogen type with outcomes of children encountering community-acquired pediatric septic shock. Pediatric Crit Care Med.

[16] Gupta S, Sakhuja A, Kumar G, McGrath E, Nanchal RS, Kashani KB (2016;). Culture-negative severe sepsis: Nationwide trends and outcomes. Chest.

[17] Blanco J, Muriel-Bombín A, Sagredo V, Taboada F, Gandía F, Tamayo L (2008;). Incidence, organ dysfunction and mortality in severe sepsis: A Spanish multicentre study. Crit Care.

[18] Cohen J, Brun-Buisson C, Torres A, Jorgensen J (2004;). Diagnosis of infection in sepsis: An evidence-based review. Crit Care Med.

[19] Li Y, Guo J, Yang H, Li H, Shen Y, Zhang D (2021;). Comparison of culture-negative and culture-positive sepsis or septic shock: A systematic review and meta-analysis. Crit Care.

[20] Garnacho-Montero J, Garcia-Garmendia JL, Barrero-Almodovar A, Jimenez-Jimenez FJ, Perez-Paredes C, Ortiz-Leyba C (2003;). Impact of adequate empirical antibiotic therapy on the outcome of patients admitted to the intensive care unit with sepsis. Crit Care Med.

[21] Harbarth S, Garbino J, Pugin J, Romand JA, Lew D, Pittet D (2003;). Inappropriate initial antimicrobial therapy and its effect on survival in a clinical trial of immunomodulating therapy for severe sepsis. Am J Med.

[22] Gupta S, Sakhuja A, Kumar G, McGrath E, Nanchal RS, Kashani KB (2016;). Culture-negative severe sepsis: Nationwide trends and outcomes. Chest.

[23] Yang L, Lin Y, Wang J, Song J, Wei B, Zhang X (2021;). Comparison of clinical characteristics and outcomes between positive and negative blood culture septic patients: A retrospective cohort study. Infect Drug Resist.

[24] Garcia PC, Tonial CT, Piva JP (2020;). Septic shock in pediatrics: The state-of-the-art. J Pediatr.

